# Bare Iron Oxide Nanoparticles: Surface Tunability for Biomedical, Sensing and Environmental Applications

**DOI:** 10.3390/nano9111608

**Published:** 2019-11-12

**Authors:** Massimiliano Magro, Fabio Vianello

**Affiliations:** Department of Comparative Biomedicine and Food Science, University of Padua, Agripolis-Viale dell’Università 16, 35020 Legnaro (PD), Italy; massimiliano.magro@unipd.it

**Keywords:** iron oxide nanoparticles, surface chemistry, nanotechnology, food chemistry, biomedicine, environment

## Abstract

Surface modification is widely assumed as a mandatory prerequisite for the real applicability of iron oxide nanoparticles. This is aimed to endow prolonged stability, electrolyte and pH tolerance as well as a desired specific surface chemistry for further functionalization to these materials. Nevertheless, coating processes have negative consequences on the sustainability of nanomaterial production contributing to high costs, heavy environmental impact and difficult scalability. In this view, bare iron oxide nanoparticles (BIONs) are arousing an increasing interest and the properties and advantages of pristine surface chemistry of iron oxide are becoming popular among the scientific community. In the authors’ knowledge, rare efforts were dedicated to the use of BIONs in biomedicine, biotechnology, food industry and environmental remediation. Furthermore, literature lacks examples highlighting the potential of BIONs as platforms for the creation of more complex nanostructured architectures, and emerging properties achievable by the direct manipulation of pristine iron oxide surfaces have been little studied. Based on authors’ background on BIONs, the present review is aimed at providing hints on the future expansion of these nanomaterials emphasizing the opportunities achievable by tuning their pristine surfaces.

## 1. Introduction

The blossoming of nanoscience and nanotechnologies has raised increasing expectations in the fields of research and industry. By definition, nanotechnology concerns the synthesis, characterization and/or the use of materials, devices or structures displaying at least one dimension (or containing components with at least one dimension) comprised in the 1–100 nm range. When a particle size stands within these limits, its physical and chemical properties significantly differ from the corresponding bulk material. Indeed, if the latter has constant physical properties regardless its size, approaching the nanoscale the same material displays drastically variable physico-chemical properties. At the nanosize, the fraction of atoms on the material surface is increasingly relevant and properties become heavily dependent on the size, the shape, the surface morphology and on several other parameters, which are still object of intense study. Thus, the state of the art is continuously enriched by the hardly predictable and fascinating properties originated by creating novel nanomaterials, and this leads to a limitless landscape of opportunities as well as challenges.

In this view, metal oxides nanoparticles have gained particular interest as their magnetic, electronic and chemical features can be modulated by a wide choice of innovative synthetic methods [1]. As examples, the electrical properties of In_2_O_3_, SnO_2_ and WO_3_, nanoparticles were exploited for gas sensing applications [2]. TiO_2_ nanoparticles were applied in photocatalysis for pollutant elimination, for the development of solar cells [3]. ZnO, thanks to its intrinsic properties as wide bandgap semiconductor, was object of extensive studies and was proposed for the development of solar cells, laser sources, gas sensors and as catalysts for various organic molecules [4]. Furthermore, one-dimensional (1-D) ZnO found application in electronics and optoelectronics [5]. GeO_2_ nanoparticles demonstrated potential applications for improved optical fibers and other optoelectronic purposes [6]. Ga_2_O_3_ nanoparticles were employed in surface-catalyzed systems for electronic or optical applications [7] CuO nanoparticles were used as redox catalysts for several oxidation processes in photothermal and photoconductive applications [8]. MgO nanoparticles were largely applied for eliminating gaseous pollutants and as a catalysts for organic syntheses [9]. Al_2_O_3_ was employed as a support for immobilizing catalysts [10]. ZrO_2_ nanoparticles are exploited as catalysts and gas sensing materials [11]. CeO_2_ nanoparticles were applied for electrochemistry, gas sensing and material chemistry [12]. Iron oxide nanomaterials (Fe_2_O_3_ and Fe_3_O_4_) were recently employed in the development of electrochemical sensors for detecting drugs, such as paracetamol [13], chloramphenicol [14] and linagliptin [15].

Nowadays, nanotechnology can count on the evolution of novel synthetic processes aimed at the fine control of composition, size, shape, surface coating and surface charge of nanomaterials to cope with complex technological tasks [16]. Among nanomaterials, magnetic nanoparticles were widely employed and in the last decades these nanostructures have been applied for the immobilization of proteins and enzymes [17], bioseparations [18], immunoassays [19], biosensors [20] and drug delivery systems [21]. In this view, it should be mentioned that magnetic nanoparticles were classified as medical devices, and they should conform to ISO 10,993 guidelines according to the US-FDA. Accordingly, some magnetic nanoparticles have been already approved for clinical MRI applications (Feridex by AMAG Pharmaceuticals, Inc., Lexington, MA, USA; Endorem by Guerbet, Villepinte, France). Among magnetic nanoparticles, iron-oxide nanoparticles (IONPs) combine many advantageous properties, such as superparamagnetism, high values of saturation magnetization, easy control by low intensity magnetic fields, as well as non-toxicity, biodegradability and biocompatibility. IONPs can be further discriminated into various categories, such as ultra-small superparamagnetic iron oxide (USPIO) [22], cross-linked iron oxide (CLIO) [23] and mono-crystalline iron oxide nanoparticles (MIONs) [24].

Maghemite (γ-Fe_2_O_3_) and magnetite (Fe_3_O_4_) represent the most widely employed crystalline iron oxide structures, finding countless applications in many fields [25], spanning from magnetic data storage [26] and pigment production [27] to electrochemistry [28] and many others. These iron oxide nanoparticles can be synthesized by different routes, even if the production of monodisperse populations of magnetic grains and the maintenance of the colloidal nature of their dispersions is still hardly achievable [29]. Indeed, generally, magnetic nanoparticles require to be coated by organic polymers or inorganic shells for their stabilization in order to prevent particle aggregation and to preserve long-term stability, electrolyte and pH tolerance and to provide a proper surface chemistry for further functionalization [30]. As an example, lipophilic drugs can easily be loaded on iron oxide nanoparticles coated with hydrophobic polymers, which release the drug when the coating degrades into the organism [31]. Notwithstanding, many of the reported polymeric coatings used for nanoparticle stabilization suffer of physical-chemical lability and eventually desorb in the bulk solution, thus reducing the stability of the resulting dispersions. Furthermore, processes proposed to coat nanoparticles are cumbersome, time-consuming, and expensive, with low yields, limiting their large scale application. At last, nanoparticle coverage reduces the average magnetic moment by introducing a diamagnetic shell in the final nanomaterial.

A number of approaches were developed for endowing nanoparticle surfaces of proper chemical functionalities. For simplicity, they can be grouped into two main categories: covalent conjugation and physical interactions. Covalent strategies involve linkages of the moiety of interest directly to amino, carboxyl, thiol or hydroxyl functional groups on the surface of previously coated magnetic nanoparticles. Generally, methods require mild reactive conditions for binding, and hence, they are suitable for organic molecules with the tendency to degradation and denaturation. Physical interactions, such as electrostatic, hydrophilic/hydrophobic and affinity interactions, can also lead to stable conjugates [32]. In particular, the latter offers the most stable noncovalent binding [33,34].

Notwithstanding that in the last decades engineered iron oxide nanomaterials have led to exciting developments and were widely employed in interdisciplinary studies involving physics, chemistry, biology and medicine, bare iron oxide nanoparticles are (BIONs) are object of an increasing interest due to advantages as cost-effectiveness and environmental sustainability. In this view, even if scientific literature on BIONs is not very diffused yet, the proposed applications are multifaceted and range from catalysis to protein purification [35,36,37]. It is important to mention that a considerable body of work is emerging from the use of BIONs in microalgae harvesting for biomass exploitation purposes [38,39,40,41,42,43,44] and from their employment in water remediation [45,46]

BIONs couple well-known features, such as magnetism, with unique properties due to the availability of under-coordinated iron(III) sites on their surfaces as a consequence of dangling bonds derived from crystal truncation. Significantly, iron(III) sites possess multifaceted reactivity. As an example, they can display catalytic activity. Hence, the catalyst can be easily driven by an external magnet. Furthermore, surface iron(III) sites behave, in some extents, as iron(III) ions providing a competitive alternative to immobilized metal affinity chromatography (IMAC) for protein purification. The knowledge of the reactivity of iron oxide surface and the comprehension of its chemical behavior may open new avenues for challenging opportunities.

Therefore, the present review, besides being aimed to the valorization of BIONs, is aimed at highlighting their importance as building blocks for producing complex nanostructured architectures, as well as of the possible achievements obtainable by tuning pristine iron oxide surfaces without the screening of stabilizing coatings.

For simplicity, scientific publications focused on innovative applications of BIONs were grouped and reviewed into the following categories: BIONs for food industry, BIONs for biomedicine, BIONs for the environment.

## 2. BIONs for Food Industry

Nanotechnology based solutions are used in nearly all the segments of the food industry, ranging from agriculture (e.g., pesticides, fertilizers and vaccines delivery, plant and animal pathogen detection and targeted genetic engineering) to food processing (e.g., flavors encapsulation or odor enhancers, food textural or quality improvement, novel gelation or viscosity control agents) to food packaging (e.g., pathogen protection, gas sensors, UV-protection films) to nutrient supplements (e.g., nutraceuticals with high stability and bioavailability). In this view, iron oxide nanoparticles presenting peculiar surface chemistries are suitable for being competitive alternatives to common processes.

Innovative analytical techniques are continuously proposed to control critical steps of an increasing variety of processes and in very different environments [47]. Nano-biosensors developed from the combination of nanotechnology and biological molecules have been proposed as effective alternatives to traditional methods in terms of sensitivity and response time [48,49].

The main advantages of using BIONs for the development of biosensors are related to the immediacy of construction. Firstly, these nanoparticles possess versatile surfaces, prone to bind macromolecules by simple self-assembly and, therefore, the resulting nano-bioconjugate is enzymatically active. Conversely, common protein immobilization processes require complicated chemical reactions, which may affect the preservation of the biological activity of the macromolecules. In addition, BIONs can also count on the electro-catalytic and magnetic properties of their iron oxide core, allowing the development of multi-functional devices.

Among proposed analytical devices, electrochemical detection is a popular method for biosensors with possible applications in food industry. Electrochemical sensors arouse interest for their convenient instrumental setup, low cost, short analysis time and experimental simplicity. Compared to other analytical methods, the electrochemical approach may be useful for food samples because matrix problems from the various food components can be avoided. Among electrochemical devices, amperometric biosensors operate by applying a constant potential at the working electrode and monitoring the current associated with the oxidation or reduction of an electroactive substance involved in the recognition process. The potential at the working electrode is maintained constant with respect to a reference electrode (usually Ag/AgCl), which is at equilibrium.

Electrochemical sensor suffers from several drawbacks, such as fouling phenomena occurring upon exposure to biological matrixes, leading to difficult redox processes at the electrode surface. In this view, electrode performances can be improved by BIONs, due to their electrocatalytic properties and high surface to mass ratio [50].

**Figure 1:**
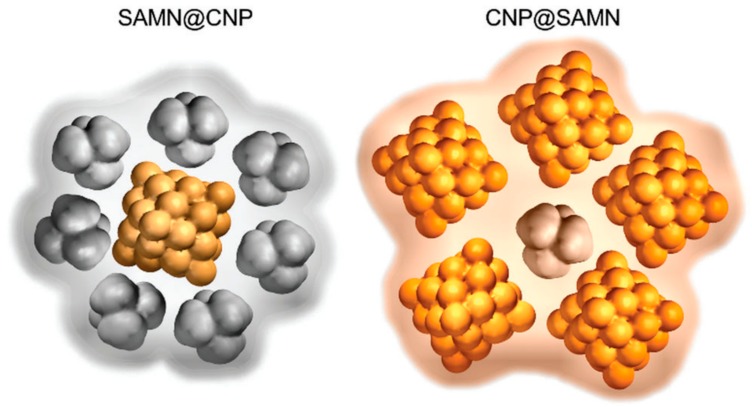
Graphical sketches of the hybrid nanomaterials constituted of BIONs and carbon nanoparticles. SAMNs (surface active maghemite nanoparticles) are a particular category of BIONs; CNPs are the carbon nanoparticles. CNPs constituted the shell (left) or the core (right) of the hybrids. Reproduced from [51], with permission from Royal Soc. Chem. 2019.

In order to improve the sensing properties and the analytical performances of biosensing devices based on nanomaterials, hybrids constituted of inorganic nanostructures and biological molecules were proposed [50]. Binary hybrids have been investigated during the past few decades due to the emerging properties of nanoparticle composites. As an example, the electrocatalytic properties of electrostatically stabilized core–shell nanoclusters composed of bare iron oxide nanoparticles (BIONs) and a set of differently charged carbon nanomaterials (Gallic acid modified carbon dots, PEG modified graphene dots and quaternized carbon dots) were investigated [51]. The combination of quaternized carbon dots (Q-CDs) with an excess of iron oxide nanoparticles led to the spontaneous formation of a Q-CD@BION hybrid, which possessed peculiar electrocatalytic properties, different from the parent components, and attributable to the influence of the strong electrostatic interactions at the interface between the carbonaceous and the inorganic nanomaterials. This led to an alteration of the iron oxide surface properties (see Figure 1). Despite Q-CDs represented only a small fraction of the hybrid (about 1% *w/w*), they were able to orient BIONs electrocatalysis toward the selective oxidation of polyphenols at low applied potentials (+0.1 V vs. SCE). The Q-CD@IONP hybrid was used for developing a coulometric sensor constituted of a simple carbon paste electrode inside a small volume electrochemical flow cell (1 µL) and finally used on real samples for assessing the concentration of polyphenols from plant extracts. The system responded linearly to chlorogenic acid, used as reference molecule, up to 1 mM, with a sensitivity of 224.6 ± 1.5 nC mM^−1^ and a limit of detection of 26.4 mM [51].

**Figure 2:**
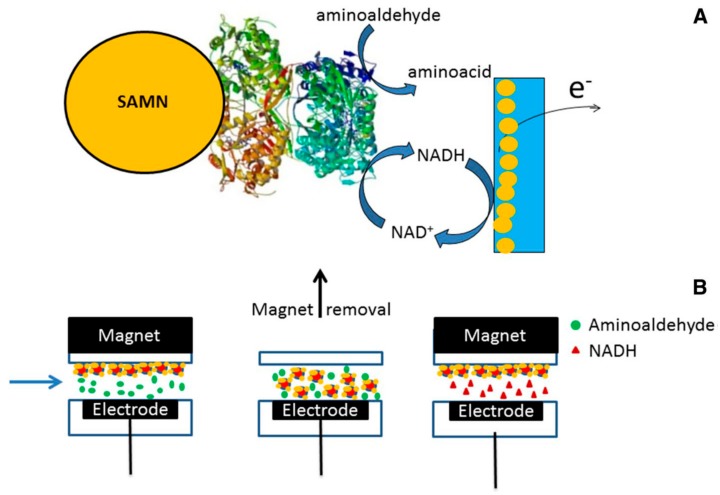
Biosensor for aminoaldehydes developed by immobilizing a recombinant aminoaldehyde dehydrogenase on peculiar BIONs (surface active maghemite nanoparticles, SAMNs). (**A**) Graphical sketch simplifying the working principle of the biosensor; (**B**) scheme representing the operational steps of the biosensor, exploiting the magnetic properties of the biological element (BION@SlAMADH1). Reproduced from [52], with permission from *Springer nature*, 2019.

The preservation of the catalytic activity of enzymes is a mandatory requirement for the real applicability of hybrid nano-bioconjugates, and the control over protein–nanoparticle interactions remains a crucial task. Actually, the creation of stable and catalytically active nano-hybrids is at the core of the development of the next generation biosensing platforms. In this view, peculiar BIONs (Surface Active Maghemite Nanoparticles, SAMNs) represent an attracting option. In fact, these nanoparticles possess the ability to selectively bind proteins and, at the same time, they display peculiar electrocatalytic properties. As an example, recombinant aminoaldehyde dehydrogenase from tomato (SlAMADH1) was used and successfully bound on BIONs by self-assembly in a proof-of-concept study [52]. Significantly, the enzymatic activity of the biomolecule was preserved for more than 6 months, and the hybrid nanomaterial (BION@SlAMADH1) was applied for the development of an electrochemical biosensor for the assessment of aminoaldehydes in commercial alcoholic beverages (Figure 2). The linearity range spanned from 25 μM to 2.0 mM aminoaldehydes, the sensitivity was 5.33 μC μM^−1^ cm^−2^, and the limit of detection (LOD) was 750 nM [52].

Alternatively, BION surface can be tuned, via simple self-assembly reactions in water, to expand the spectrum of developable biosensing systems. In this view, rhodamine isothiocyanate (RITC) and tannic acid (TA) were used as bridging functionalities for the immobilization of glucose oxidase and laccase, and the as-obtained nano-bioconjugates were successfully exploited for building electrochemical biosensors for the detection of glucose in apricot juice (0–1.5 mM linearity range, 45.85 nA mM^−1^ cm^−2^ sensitivity and 0.9 µM LOD) [53] and polyphenols in blueberry extracts (100 nM-50 μM linearity range, 868.9 ± 1.9 nA μM^−1^ sensitivity and 81 nM LOD) [54].

The determination of foodborne pathogens is obviously a crucial task in food industry. Traditional diagnostic techniques for pathogens comprise colony counting, immunological-based methods and polymerase chain reaction (PCR), but all these methods present some important drawbacks [55]. Most of them need expensive, sophisticated instrumentation which requires trained personnel; hence, they are hardly suitable for developing countries. Their application use is also limited in the field, such as distribution centers.

Moreover, the short shelf-life and the high cost of some reagents, such as DNA primers and enzymes, represent a limit for the application of most conventional detection techniques for pathogens. Furthermore, notwithstanding their sensitivity and reproducibility, current technologies, such as ELISA and PCR, require complex sample preparation procedures and count on long readout times, which delay prompt responses and disease containment.

**Figure 3:**
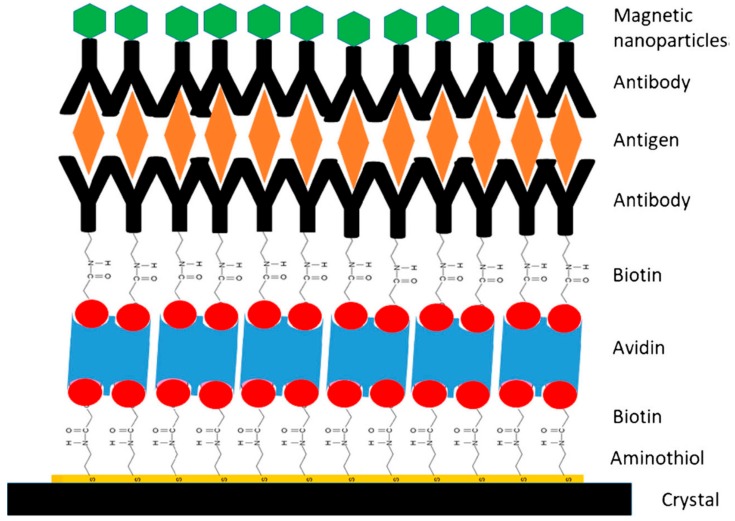
Scheme of a modified piezoelectric crystal for the detection of *Listeria monocytogenes* after magnetic capturing by SAMN@avidin@Anti-*Lm*. Reproduced from [57], with permission from Springer Nature, 2019.

The detection of bacteria can be obtained by mass-sensitive biosensors, in which the transduction is due to small changes of the mass of the sensing material. Mass measurements are generally performed by piezoelectric crystals, which oscillate at a specific frequency according to the application of a periodic electric signal. Therefore, when the mass increases due to the binding of an analyte (the pathogen), the frequency of oscillation of the crystal decreases and this frequency change can be used to determine the additional mass on the crystal. When a piezoelectric sensor surface coated with an antibody is placed in a solution containing the pathogen, the recognition of the bacteria by the antibody on the detecting surface results in an increase in the crystal mass, leading to a corresponding oscillation frequency shift. Magnetic nanoparticles, properly functionalized with antibodies, can be used for the fast testing of the presence of pathogens in foods by capturing and magnetically concentrating the microorganisms on the sensing device [56]. This leads to the amplification of the detectable signal. Nevertheless, even if the production of custom immuno-magnetic devices would be strongly preferred at the laboratory bench, nanoparticle engineering is still a prerogative of manufacturers. This is due to the chemical complexity of most of the common antibody immobilization protocols, as well as the need of specific competences from the user. Therefore, BIONs were proposed as versatile platform for developing tailored immuno-magnetic devices by very simple wet reactions. Significantly, this opportunity was provided by the characteristic binding properties of the pristine iron surface. Both native and biotinylated antibodies were conjugated to nanoparticles and the as-obtained multifunctional nanodevices were successfully tested for the recognition of *Campylobacter fetus* and *Listeria monocytogenes* by a piezoelectric quartz crystal microbalance (QCM) sensor (Figure 3) [57]. The system was applied for the detection of *L. monocytogenes* in milk, showing a detection limit of 3 bacterial cells per sample (200 μL).

Beyond analytical purposes, iron oxide nanomaterials were also used for the study of the metabolism of foodborne pathogens. As an example, some strains of *Pseudomonas fluorescens* produce a characteristic blue pigment that was recently investigated on cheeses for the negative economic impact on dairy industry [58,59,60]. The chemical nature of the pigment responsible for the blue discoloration has generated an intense debate over the last years [59,61], and it was demonstrated that blue discoloration in *Pseudomonas fluorescens* is correlated to the high resistance to oxidative stress induced by hydrogen peroxide. Indeed, this property was not displayed by white mutants [62]. Conventional colorimetric methods failed to evidence any difference between the antioxidant activity of blue and white microorganisms, and in a recent manuscript the mechanism of H_2_O_2_ scavenging activity by blue strains of *P. fluorescens* was studied by a nanotechnology based electrochemical approach [63].

The control of foodborne pathogens represents an important matter of concern and efforts to reduce the possibility of disease outbreaks are constantly in progress [64]. Food industries normally use antibiotics and synthetic preservatives during food production processes to prevent this problem. However, the combination of inappropriate and excessive use of chemical substances led to heavy chemical contamination of foods and to the emergence of drug resistant bacteria, increasing the difficulties of controlling the proliferation of foodborne diseases. In this view, iron oxide nanoparticles were modified with tannic acid, and the resulting core-shell hybrid nanomaterial resulted as one of the most robust tannic acid complexes to date [65]. Indeed, a drastic reorganization of the crystalline structure of the nanoparticle at the boundary with the solvent was observed, leading to the formation of a ferric tannate network that revealed antimicrobial properties. Thus, the core-shell hybrid nanomaterial was tested on *Listeria monocytogenes* showing a bacteriostatic effect. Moreover, the tannic acid modified nanostructure combined the inhibitory activity toward *L. monocytogenes* to the opportunity of being magnetically removed from the food matrix, hence leaving no residues in the final food products. The system was proposed as an effective, low-cost and environment friendly processing aid for the surface treatment in food industry [66].

**Figure 4:**
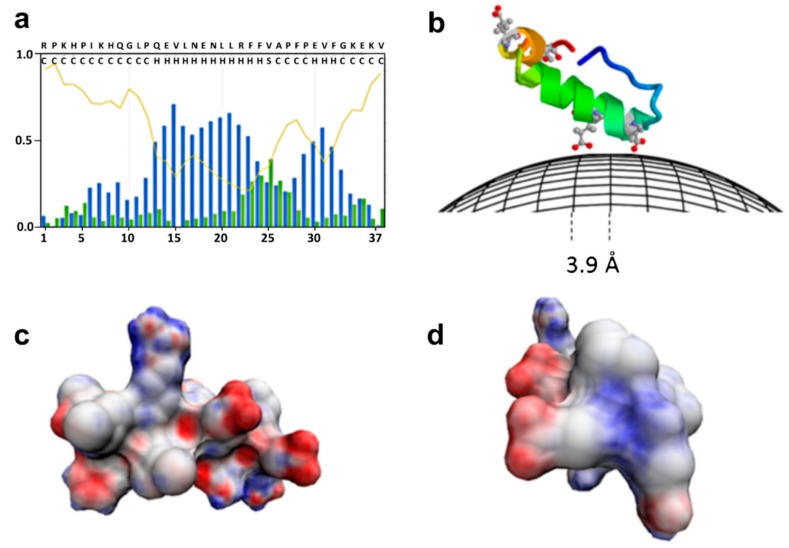
Binding of a milk peptide (αs1-casein 1-37)) on the surface of BIONs. (**a**) Statistical populations of ordered and disordered regions for the peptide according to the prediction of the s2D method. Coil curve describes the prediction of the probability to be unstructured and flexible. Blue and green bars represent the prediction of the statistical population for the helix and strand conformations; (**b**) graphical sketch of the peptide binding on BIONs where structured parts of the peptide are represented by helical models. The peptide structure was created by running QUARK algorithm; (**c**) and (**d**) helical model of the portion of the peptide predicted to be in helix according to s2D, two different views. The negatively charged surface is highlighted in red and computed with a Poisson–Boltzmann electrostatics calculations by 3D-HM. Reproduced from [69], with permission from Springer Nature, 2019.

The analysis of protein corona on nanomaterials represents the last frontier in proteomics, offering an alternative diagnostic strategy aimed at revealing faint proteome alterations correlated to a specific sample modification. Upon exposure to a biological matrix, nanomaterials are subjected to the spontaneous formation of a stable protein coating defined “protein corona” [67]. This common phenomenon leads to the formation of a protein shell, whose composition changes according to the proteome variations following, for example, a disease occurrence. Thus, the nanomaterial can be used as a probe for fishing specific peptides or proteins constituting a protein corona in a biological sample, simplifying the whole sample proteome [68]. This innovative approach was adopted for developing a fast analytical procedure for testing milk quality. MALDI-TOF mass spectrometry evidenced a hidden biomarker in the protein corona on nanoparticle surface, enabling the discrimination between milk coming from healthy and mastitis affected bovines (Figure 4) [69].

Another matter of concern for food industry is the large volume of residual biomass generated by the large-scale purification of natural compounds. This heavily influences processing industries, logistic sizing, magnitude of solvent consumption employed for extraction processes and the overall chemical and biological waste generation. In order to cope with this issue and, more in general, to move the applications of magnetic nanoparticles for the isolation and purification of biomolecules to an industrial level, an automatic modular pilot system was developed and applied for the continuous magnetic purification of curcuminoids, which are natural compounds of high nutraceutical value, from *Curcuma longa* roots. This novel magnetic separation device demonstrated the scalability of the purification processes by magnetic nanoparticles and embodies the fundamental principles of sustainable innovation [70].

Similarly to the presented purification of curcumin from *Curcuma longa* rhizome extracts and based as well on the specific iron binding properties of the molecule of interest, BIONs are a promising option for overcoming conventional large scale elimination of toxic substances from food matrices, such as citrinin, a nephrotoxic mycotoxin that can be synthesized by *Monascus* mold during the fermentation process in foods [71].

## 3. BIONs for Biomedicine

In the last decades nanotechnology provided a wide range of new materials to biomedicine. As examples, carbon based nanomaterials, such as carbon nanotubes, fullerenes, graphene, nano-diamonds and fluorescent carbon quantum dots (CQDs) were exploited in a variety of biomedical applications [71]. CQDs, due to their advantageous chemical-physical features such as optical and fluorescence characteristics, aroused increasing interest and led to applications in biosensing and bioimaging [72]. Gold nanoparticles (AuNPs) appear among the most diffusely applied nanomaterials and are generally used after proper surface coating. The surface coating, its nature and structural organization, dominates the nanoparticle interactions with biological systems, influencing nanoparticle biodistribution and effects in biological systems. Surface coatings on AuNPs, generally by thiol containing molecules, may bear different functional groups, such as carboxylate, phosphate or sulfate. These nanosystems were extensively studied and applied in biomedicine, including for the development of biosensors, immunoassays, phototherapy, optical bioimaging, tracking of cells, targeted delivery of drugs [73]. Notably, toxicity limits the applications of AuNPs in clinical therapy. Nanomaterials based on polymeric nanoparticles were developed to improve the diagnosis and treatment of a wide range of diseases, spanning from viral infections, cardiovascular diseases and cancer to pulmonary and urinary tract infections [74]. Polymeric nanoparticles attracted the interest of the scientific community due to their structural versatility. In fact, they can be easily tuned to load and deliver their payload to the desired site of action and to respond to specific external or physiological stimuli [75]. The chemistry of polymeric nanoparticles and of their cargo influences their biocompatibility, stability, biodegradability, as well as their biodistribution, cellular and subcellular fate. Silica nanoparticles (SiO_2_) are not endowed with particular chemical-physical properties: Nevertheless, they found applications in biomedicine as versatile tools for designing nanosized probes and carriers [76]. Indeed, silica nanoparticles possess a well-defined and easily tunable surface chemistry, which can be modified with different functionalities and linked to different biomolecules, enabling the fine control of the interplay with biological entities [77]. Mesoporous silica nanoparticles, characterized by pore sizes ranging from 2 to 50 nm, were excellent options for drug delivery and biomedical applications [78]. The mesoporous structure allows the control of drug loading and the control of release kinetics, enhancing drug therapeutic efficacy and reducing the toxicity [79]. The design of functionalized silica nanoparticles focused on the selective delivery of drugs and radionuclides to improve diagnosis and treatment were highlighted by the current progresses in the field of pharmaceutical nanotechnology [80].

**Figure 5:**
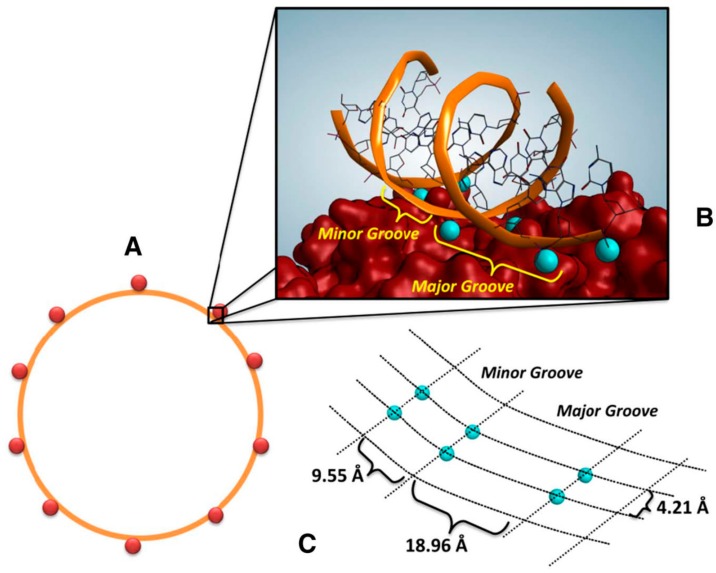
Schematic representation of the interactions between plasmidic DNA and BIONs. (**A**) One molecule of plasmidic DNA (in orange) binds about 10 nanoparticles (in red); (**B**) Interactions between Fe^3+^ (in blue) and DNA on the surface of BION; a possible representation of the nanoparticle surface is reported. (**C**) Projection of Fe^3+^ (in blue) on BION surface, in which the distances among Fe^3+^ are shown. Reproduced from [87], with permission from Elsevier, 2019.

The broad spectrum of favorable properties of iron oxide nanoparticles encouraged their utilization for diagnostic and therapeutic applications in biomedicine, and some were already accepted for clinical purposes or expected for new approved issues in the near future [81]. Gene therapy, cell imaging and tissue regeneration are among the most challenging biomedical applications of iron oxide nanoparticles. Indeed, BIONs are characterized by surface tunability, cost-effectiveness and biocompatibility, which make these pristine nanomaterial an attracting option in biomedicine. Moreover, BIONs can be further derivatized to be upgraded to multifunctional nano-devices for theranostic uses. Indeed, engineered BIONs were developed to meet the increasing interests for in vivo imaging, gene delivery, drug targeting and biomarker detection. As an example, a novel multifunctional nano-immunosensor coupling the magnetic properties of nanosized iron oxides and the optical features of gold nanoparticles modified with specific aptamers was developed and tested for the recognition of dengue virus [82]. The proposed complex nano-architecture allowed the rapid visual detection of the virus by a simple colorimetric assay. It should be considered that the development of this hybrid nanomaterial was extremely facilitated by the use of BIONs. Indeed, chemical approaches commonly employed for manipulating coated magnetic nanoparticles are considerably more complicated, requiring solvents, expensive chemicals, drastic conditions and trained operators. Alternatively, iron oxide nanoparticles can be considered as intelligent, universal nano-vectors, able to match drug protection (avoiding drug loss), due to the unusual specific nature of the surface interactions, with a pronounced reactivity. This, along with the extensively demonstrated colloidal stability, excellent cell uptake [83], stability in the host cells, low toxicity and great MRI contrast agent properties [84], makes iron oxide nanoparticles an elective tool for biomedical applications. These properties, in combination with the ones provided by surface ligands led to promising multifaceted nanodevices. Long-term dual imaging (fluorescence and MRI) nanoprobes were obtained by the direct immobilization of rhodamine isothycianate on BIONs and demonstrated in vitro on mesenchymal stromal cells [85]. The biological properties of curcumin were endowed to BIONs creating a magnetically drivable nanovehicle with antioxidant properties [86]. Furthermore, a plasmid (pDNA) harboring the coding gene for GFP was directly chemisorbed onto iron oxide nanoparticles, leading to a novel DNA nanovector that was successfully internalized and stored into mesenchymal stem cells. Transfection by the proposed system occurred and GFP expression was higher than that observed with lipofectamine procedure, even in the absence of external magnetic field (Figure 5) [87].

As above mentioned, the combination of different nanomaterials represents a challenge for the emerging properties and features of the resulting nano-hybrids. As an example, an electrostatically stabilized ternary hybrid, constituted of a core of BION@DNA coated by spermidine based quantum dots (Spd-CQDs), was developed, fully characterized and tested on HeLa cells in order to evaluate its cellular uptake and biocompatibility. This novel ternary nano-hybrid with multifaceted properties, ranging from superparamagnetism to fluorescence, represents a novel option for cell tracking [88]. Moreover, iron oxide nanoparticles were used for biomarker identification and detection by immobilizing different biomolecules, such as antibodies [89], aptamers [90] and enzymes [91] or peptide fragments, such as RGD [92] or polysaccharides [93].

**Figure 6:**
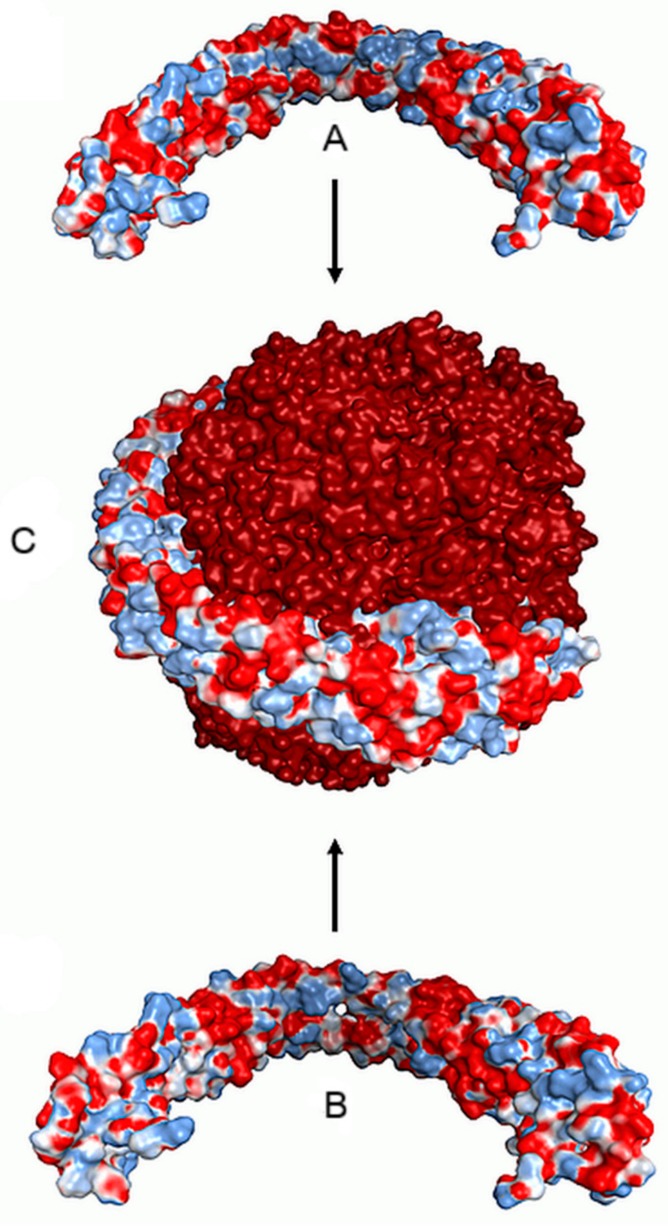
Pictorial representation of the binding on an iron oxide nanoparticle of a dimer of apolipoporotein A1 from gilthead bream (*Sparus aurata*). A dimer of apolipoporotein A1 (A, B) is shown as Connolly’s electrostatic surface charge distribution, by AMBER99 force field. Blue color indicates positive charges and red color indicates negative charges. Adapted from [95], with permission from A.C.S., 2019.

The potential of bare iron oxide nanoparticles as antibiotic carriers for the treatment of specific vertically transmissible infections in fishes, such as those affecting farmed trouts caused by bacteria (such as *Flavobacterium psychrophilum* and *Bacterium salmoninarum*), was explored in zebrafish (*Danio rerio*) by providing a drug nanovehicle directly into the farming water [94]. The ability of bare iron oxide nanoparticles to circumvent the clearance by the immune system was examined in vivo using the same animal model. Iron oxide nanoparticles overcame the intestinal barrier and led to the prolonged (28 days) and specific organotropic delivery of the antibiotic to the fish ovary. Significantly, no adverse effects were observed. Interestingly, a structural analogy between high-density lipoproteins (HDL) and the complex formed between iron oxide nanoparticles and apolipoprotein A1 (Figure 6) suggests that biomimetic properties play an important role in such a complex phenomenon [95].

The presence of a specific proteome in a living organism can represent a crucial gap between in vivo and in vitro testing of nanomaterials. This aspect was recently evidenced by using fish organ cultures and testing different oxytetracycline exposure methods, including antibiotic bound on BIONs. Notably, the exposure of an isolated organ to BIONs bearing the antibiotic cannot be representative of the complexity of the interactions occurring in a whole organism [96].

## 4. BIONs for the Environment

A deep analysis of the potential environmental risks associated with the intensive extensive use of iron oxide nanoparticles cannot be further delayed, and even the factors related with epigenetic phenomena and long-term effects should be assessed. Iron oxide nanoparticles, due to their wide utilization for research purposes and perspectives in clinical practice, represent an ideal model for proposing an acceptable and comprehensive platform for the punctual classification of environmental risks of nanotechnology. In fact, the identification and definition of general protocols for the assessment of nanomaterial safety toward environmental protection and human health and the evaluation of long-term effects of nanoparticle drugs complexes and contrast agents for medical applications are of primary interest [97].

**Figure 7:**
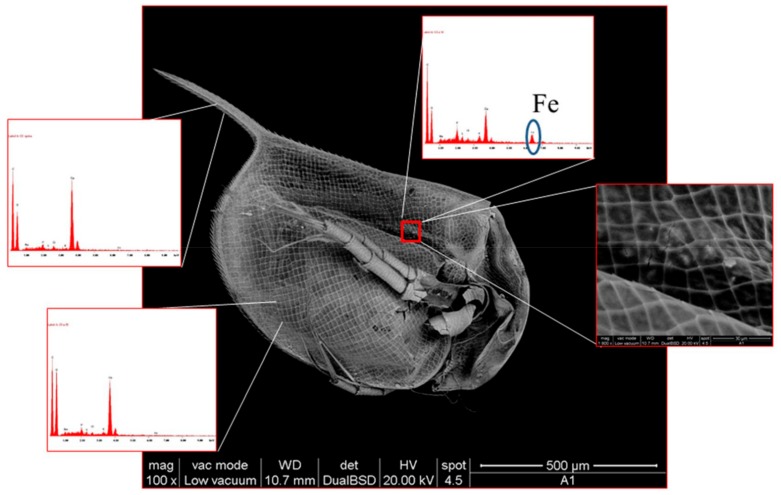
Micrograph by scanning electron microscopy of Daphnia Magna after incubation in a solution containing 1.25 mg L^−1^ BIONs. Insets: Elemental analysis by energy dispersive X-ray spectroscopy and higher magnification of gut detail. Reproduced from [98], with permission from Springer Nature, 2019.

It is recognized that the effects of nanomaterials on biological systems must be faced with interdisciplinary approaches and the coordinated contributions of chemists, physicists, biologists, pharmacologists and clinical doctors. On these bases, an eco-toxicological study on iron oxide nanoparticles was carried out using *Daphnia magna* as animal model (Figure 7).

The surface reactivity of the nanomaterial emerged as a fundamental issue for determining its effects on biological systems, hence tests on acute and chronic toxicity were compared to nanoparticle distribution into the crustacean, intake/depletion rates and swimming performances. Fast depuration from the nanomaterial and absence of delayed effects indicated no retention and good tolerance within the organism, hence substantiating the safety of iron oxide nanoparticles as a tool for aquaculture purposes [98].

**Figure 8:**
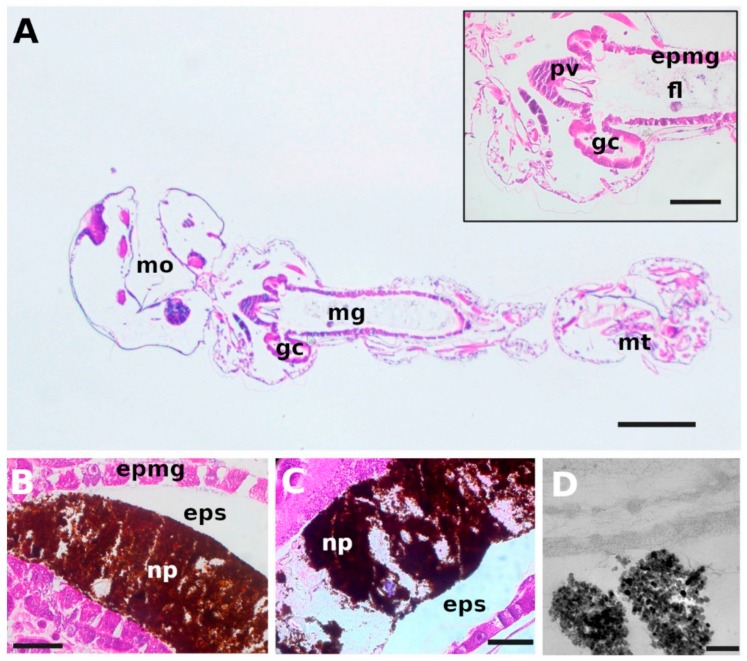
Microscopic characterization of *Aedes aegypti* larvae. (**A**) Optical micrograph of the sagittal section of a control sample. Inset: Optical micrograph of the section of gastric caeca, proventriculus and anterior end of mid-gut. (**B**) Optical micrograph of the midgut of a larva after exposure to 100 mg L^−1^ BIONs for 1 h, evidencing the accumulation of nanoparticles in the gut lumen. (**C**) Optical micrograph of the midgut of a larva exposed to 100 mg L^−1^ BION@chlorin for 80 min. (**D**) TEM micrograph of the gut of a *Aedes aegypti* larva exposed to 100 mg L^−1^ BION@chlorin for 1 h. (epmg), epithelium of the midgut; (eps), ectoperitrophic space; (fl), food in gut lumen; (gc), gastric caeca; (mg), midgut; (mo), mouth; (mt), malpighian tubules; (pv), proventriculus; (np), nanoparticles. Size bars: A = 250 μm, inset = 100 μm, B and C = 50 μm, D = 200 nm. Reproduced from [99], with permission from Elsevier, 12019.

In order to cope with the worldwide threat of diseases vectored by mosquitos, novel solutions aimed at the enhancement of the control over these detrimental insects are strongly desired. In this view, systems coupling low environmental impacts with high insecticidal activity represent a crucial goal nowadays, particularly in the view of their application in aquaculture. An eco-friendly photosensitizing magnetic nanocarrier with larvicidal effects on *Aedes aegypti* was proposed [99]. A core-shell nanocarrier was synthesized, combining iron oxide nanoparticles and chlorin-e6. The photosensitizing functionality was provided to the magnetic core by a simple wet reaction, exploiting the chelating groups of the organic molecule. The self-assembled hybrid possessed several advantageous properties, such as magnetic drivability, physico-chemical robustness and high colloidal stability. This last feature is a mandatory prerequisite for the application in aquatic systems. Most importantly, the proposed photosensitizing nano-device presented real chances to be a competitor of conventional insecticides, considering its high efficiency, as demonstrated on larvae of *Aedes aegypti*, and its minimal environmental impact (Figure 8) [99].

Magnetic separation strategies by nanomaterials represent competitive options or, at least, valuable complementing methods to traditional approaches for the removal of organic and inorganic pollutants in water. Magnetic nanoparticles have been demonstrated to be effective, rapid and cost-effective tools for water remediation issues [100,101,102,103]. Separation processes based on magnetic particles offer the advantage of eliminating absorbed toxic compounds by the application of an external magnetic field. In this context, iron oxide nanoparticles represent the gold standard.

Water pollution from antibiotics is a global threat due to their extensive and durable presence in the ecosystems and their bulky use [104]. Indeed, the use of antibiotics for human and veterinary medicine can be estimated to overcome 100 ktons per year worldwide [105], and most of these compounds in the environment are very stable, exerting for long time their activity, thus granting their effects for long [106]. In this context, the thermodynamically favorable complexation chemistry of different iron oxides, such as magnetite, goethite or hematite, [107,108] have the potential for representing efficient sorbents for antibiotics. In fact, many of these molecules are strong chelating agents. Nevertheless, despite the promising possibilities offered by the surface complexation chemistry of iron oxides, only rare efforts were dedicated to the use of bare iron oxide nanoparticles for water remediation from antibiotics, such as oxytetracycline [107,109]. This is not surprising as the preparation of stable colloidal suspensions of bare metal oxides remains a challenge for preparative nanotechnology [110]. A novel category of BIONs was employed for the removal of oxytetracycline in large water volumes where, in order to simulate a real in situ scenario, a population of zebrafish (*Danio rerio*) was introduced. The introduction of the nanomaterial in water led to the complete elimination of the antibiotic without any sign of toxicity for the animal model. Furthermore, according to a toxicological characterization on oxytetracycline sensitive *Escherichia coli* strains, the entrapped antibiotic resulted biologically safe. Thus, the proposed nanomaterial emerged as a competitive option for water remediation from oxytetracycline and, in particular, for in situ applications [111].

Peculiar BIONs were also proposed for ex situ and in situ water remediation of Cr(VI) pollution. This nanomaterial was proposed as suitable sorbent for entrapping and recovering dissolved chromium in water. Significantly, the BION@CrVI complex showed no genotoxicity on a chromium sensitive strain of *Salmonella typhimurium TA100* [112].

## 5. Overall Conclusions and Perspectives

Iron oxide nanomaterials are already applied by a number of high-tech industries due to their outstanding magnetic, catalytic and electrochemical properties, as a function of their size, structure, shape and surface chemistry. Indeed, iron oxide nanoparticles, due to their structural peculiarities, were applied for developing novel platforms for biomedical, food safety and environmental issues, and efficient promising solutions to overcome the limits and drawbacks of currently available techniques were proposed. As examples, novel user-friendly, sensitive and cheap diagnostic assays to assist the control of microbial pathogens and food animal diseases were developed [113,114]. Furthermore, bare iron oxide nanoparticles offer the opportunity of covalently interacting with ligands, allowing the development of robust delivery tools. Therefore, magnetic drivable gene and drug nano-vectors can be produced [87,95,115].

In the near future, a wide scenario of innovations is promised by the novel physicochemical properties of the hybrids involving these nanomaterials, generating a constantly increasing number of innovative applications.

Among proposed nanodevices, one of the most promising achievement relies on the evidences that naked metal oxides can provide a stealth behavior within organisms with important in vivo applications. In fact, the “stealth effect” consists in the ability of avoiding the immune system clearance of the nanomaterial. It should be considered that recognition of nanoparticles by cells was recently defined as “cell vision”, emphasizing the concept of what the cell “sees” when it faces nanoparticles [116]. In this context, the formation of a shell of biologically recognizable proteins could play a fundamental role for enhancing nanoparticle internalization by specific cells and tissues [117]. In fact, the protein “corona”, shaping surface properties, charges, hydrodynamic size and resistance to aggregation, largely defines the biological identity of the particle [118]. Exerting control over protein-nanoparticle interactions is a problematic task, considering that the binding of enzymes and proteins on nanoparticle surfaces leads to often unavoidable unspecific interactions [119,120]. Contrary to recent findings reporting on the loss of targeting capabilities of nanoparticles upon the spontaneous formation of a protein corona [121,122], some iron oxide nanoparticles are able to select few proteins within a biological medium, forming stable nano-bioconjugates, which are easily recognizable and incorporated by cells [83]. Thus, the control of the interactions of macromolecules with nanomaterial surfaces will be a relevant aspect of future applications of iron oxide nanoparticles and possibly will be exploited for the preparation of suitably targeted nanosystems for interacting with specific tissues [123].

Finally, an important task in the future will be our improvement in the knowledge of the nature of the macromolecule-iron oxide nanoparticle interactions and their influence on the biological activity of the resulting nano-bioconjugate.

## References

[1] Chavali M.S., Nikolova M.P. (2019). Metal oxide nanoparticles and their applications in nanotechnology. SN Appl. Sci..

[2] Franke M.E., Koplin T.J., Simon U. (2006). Metal and metal oxide nanoparticles in chemiresistors: Does the nanoscale matter?. Small.

[3] Wilkes J.S., Zaworotko M.J. (1992). Air and water stable 1-ethyl-3-methylimidazolium based ionic liquids. Chem. Commun..

[4] Nuraje N., Su K., Haboosheh A., Samson J., Manning E.P., Yang N.-I., Matsui H. (2006). Room temperature synthesis of ferroelectric barium titanate nanoparticles using peptide nanorings as templates. Adv. Mater..

[5] Yahiro J., Oaki Y., Imai H. (2006). Biomimetic synthesis of wurtzite ZnO nanowires possessing a mosaic structure. Small.

[6] Margaryan A.A., Liu W. (1993). Prospects of using germanium dioxide-based glasses for optics. Opt. Eng..

[7] Lee S.-Y., Gao X., Matsui H. (2007). Biomimetic and aggregation driven crystallization route for the room-temperature material synthesis: The growth of β-Ga_2_O_3_ nanoparticles on peptide assemblies as nanoreactors. J. Am. Chem. Soc..

[8] Klem M.T., Mosolf J., Young M., Douglas T. (2008). Photochemical mineralization of europium, titanium, and iron oxyhydroxide nanoparticles in the ferritin protein cage. Inorg. Chem..

[9] Zhang W., Zhang D., Fan T., Ding J., Guo Q., Ogawa H. (2006). Fabrication of ZnO microtubes with adjustable nanopores on the walls by the templating of butterfly wing scales. Nanotechnology.

[10] Aizenberg J., Hanson J., Koetzle T.F., Weiner S., Addadi L. (1997). Control of macromolecule distribution within synthetic and biogenic single calcite crystals. J. Am. Chem. Soc..

[11] Biro L.P., Balint Z., Kertesz K., Vertesy Z., Mark G.I., Tapaszto L., Vigneron J.P., Lousse V. (2007). Photonic crystal structures of biologic origin: Butterfly wing scales. Mater. Res. Soc. Symp. Proc..

[12] Zou D., Xu C., Luo H., Wang L., Ying T. (2008). Synthesis of Co_3_O_4_ nanoparticles via an ionic liquid-assisted methodology at room temperature. Mater. Lett..

[13] Vinay M.M., Nayaka Y.A. (2019). Iron oxide (Fe_2_O_3_) nanoparticles modified carbon paste electrode as an advanced material for electrochemical investigation of paracetamol and dopamine. J. Sci. Adv. Mater. Dev..

[14] Jakubec P., Urbanová V., Medříková Z., Zbořil R. (2016). Advanced sensing of antibiotics with magnetic gold nanocomposite: Electrochemical detection of chloramphenicol. Chem. Eur. J..

[15] Manal A., Azab S.M., Hendawy H.A. (2019). A facile nano-iron oxide sensor for the electrochemical detection of the anti-diabetic drug linagliptin in the presence of glucose and metformin. Bull. Nat. Res. Cent..

[16] Xie J., Jon S. (2012). Magnetic nanoparticle-based theranostics. Theranostics.

[17] Yang H.H., Zhang S.Q., Chen X.L., Zhuang Z.X., Xu J.G., Wang X.R. (2004). Magnetite-containing spherical silica nanoparticles for biocatalysis and bioseparations. Anal. Chem..

[18] Ito A., Shinkai M., Honda H., Kobayashi T. (2005). Medical application of functionalized magnetic nanoparticles. J. Biosci. Bioeng..

[19] Tanaka T., Matsunaga T. (2000). Fully automated chemiluminescence immunoassay of insulin using antibody-protein A-bacterial magnetic particle complexes. Anal. Chem..

[20] Liu Z., Liu Y., Yang H., Yang Y., Shen G., Yu R. (2005). A phenol biosensor based on immobilizing tyrosinase to modified core-shell magnetic nanoparticles supported at a carbon paste electrode. Anal. Chim. Acta.

[21] Neuberger T., Schopf B., Hofmann H., Hofmann M., Rechenberg B.V. (2005). Superparamagnetic nanoparticles for biomedical applications: Possibilities and limitations of a new drug delivery system. J. Magn. Magn. Mater..

[22] Saito S., Tsugeno M., Koto D., Mori Y., Yoshioka Y., Nohara S., Murase K. (2012). Impact of surface coating and particle size on the uptake of small and ultrasmall superparamagnetic iron oxide nanoparticles by macrophages. Int. J. Nanomed..

[23] Tassa C., Shaw S.Y., Weissleder R. (2011). Dextran-coated iron oxide nanoparticles: A versatile platform for targeted molecular imaging, molecular diagnostics, and therapy. Acc. Chem. Res..

[24] Wang J., Huang Y., David A.E., Chertok B., Zhang L., Yu F., Yang V.C. (2012). Magnetic nanoparticles for MRI of brain tumors. Curr. Pharm. Biotechnol..

[25] Tran P.H.-L., Tran T.T.-D., Vo T.V., Lee B.-J. (2012). Promising iron oxide-based magnetic nanoparticles in biomedical engineering. Arch. Pharm. Res..

[26] Sasaki Y., Usuki N., Matsuo K., Kishimoto M. (2005). Development of NanoCAP technology for high-density recording. IEEE Trans. Magn..

[27] Fouda M.F.R., El-Kholy M.B., Moustafa S.A., Hussien A.I., Wahba M.A., El-Shahat M.F. (2012). Synthesis and characterization of nanosized Fe_2_O_3_ pigments. Int. J. Inorg. Chem..

[28] Magro M., Baratella D., Pianca N., Toninello A., Grancara S., Zboril R., Vianello F. (2013). Electrochemical determination of hydrogen peroxide production by isolated mitochondria: A novel nanocomposite carbon–maghemite nanoparticle electrode. Sens. Actuator B Chem..

[29] Laurent S., Forge D., Port M., Roch A., Robic C., Vander Elst L., Muller R.N. (2008). Magnetic iron oxide nanoparticles: Synthesis, stabilization, vectorization, physicochemical characterizations, and biological applications. Chem. Rev..

[30] Ahmed N., Fessi H., Elaissari A. (2012). Theranostic applications of nanoparticles in cancer. Drug Discov. Today.

[31] Soenen S.J., Hodenius M., De Cuyper M. (2009). Magnetoliposomes: Versatile innovative nanocolloids for use in biotechnology and biomedicine. Nanomedicine.

[32] Wei H., Insin N., Lee J., Han H.S., Cordero J.M., Liu W., Bawendi M.G. (2012). Compact zwitterion-coated iron oxide nanoparticles for biological applications. Nano Lett..

[33] Wahajuddin S.S.P., Arora S. (2012). Superparamagnetic iron oxide nanoparticles: Magnetic nanoplatforms as drug carriers. Int. J. Nanomed..

[34] Meyers S.R., Grinstaff M.W. (2012). Biocompatible and bioactive surface modifications for prolonged in vivo efficacy. Chem. Rev..

[35] Hudson R., Feng Y., Varma R.S., Moores A. (2014). Bare magnetic nanoparticles: Sustainable synthesis and applications in catalytic organic transformations. Green Chem..

[36] Schwaminger S.P., Fraga-García P., Blank-Shim S.A., Straub T., Haslbeck M., Muraca F., Dawson K.A., Berensmeier S. (2019). Magnetic one-Step purification of his-tagged protein by bare iron oxide nanoparticles. ACS Omega.

[37] Schwaminger S.P., Blank-Shim S.A., Scheifele I., Pipich V., Fraga-García P., Berensmeier S. (2019). Design of interactions between nanomaterials and proteins: A highly affine peptide tag to bare iron oxide nanoparticles for magnetic protein separation. Biotechnol. J..

[38] Fraga-García P., Kubbutat P., Brammen M., Schwaminger S., Berensmeier S. (2018). Bare iron oxide nanoparticles for magnetic harvesting of microalgae: From interaction behavior to process realization. Nanomaterials.

[39] Wang S.K., Stiles A.R., Guo C., Liu C.Z. (2015). Harvesting microalgae by magnetic separation: A review. Algal Res..

[40] Xu L., Guo C., Wang F., Zheng S., Liu C.Z. (2011). A simple and rapid harvesting method for microalgae by in situ magnetic separation. Bioresour. Technol..

[41] Lee Y.C., Lee K., Oh Y.K. (2015). Recent nanoparticle engineering advances in microalgal cultivation and harvesting processes of biodiesel production: A review. Bioresour. Technol..

[42] Safarik I., Pospiskova K., Baldikova E., Puri M. (2017). Magnetic particles for microalgae separation and biotechnology. Food Bioactives.

[43] Prochazkova G., Safarik I., Branyik T. (2013). Harvesting microalgae with microwave synthesized magnetic microparticles. Bioresour. Technol..

[44] Toh P.Y., Ng B.W., Ahmad A.L., Chieh D.C.J., Lim J. (2014). The role of particle-to-cell interactions in dictating nanoparticle aided magnetophoretic separation of microalgal cells. Nanoscale.

[45] Lin S., Lu D., Liu Z. (2012). Removal of Arsenic Contaminants with Magnetic γ-Fe_2_O_3_ Nanoparticles. Chem. Eng. J..

[46] Liu R., Liu J.F., Zhang L.Q., Sun J.F., Jiang G.B. (2016). Low temperature synthesized ultrathin γ-Fe_2_O_3_ nanosheets show similar adsorption behavior for As(III) and As(V). J. Mater. Chem. A.

[47] Rana S., Yeh Y.C., Rotello V.M. (2010). Engineering the nanoparticle protein interface: Applications and possibilities. Curr. Opin. Chem. Biol..

[48] Velusamy V., Arshak K., Korostynska O., Oliwa K., Adley C. (2010). An overview of foodborne pathogen detection: In the perspective of biosensors. Biotechnol. Adv..

[49] Hierlemann A., Gutierrez-Osuna R. (2008). Higher-order chemical sensing. Chem. Rev..

[50] Urbanova V., Magro M., Gedanken A., Baratella D., Vianello F., Zboril R. (2014). Nanocrystalline Iron Oxides, Composites, and Related Materials as a Platform for Electrochemical, Magnetic, and Chemical Biosensors. Chem. Mater..

[51] Baratella D., Magro M., Jakubec P., Bonaiuto E., de Almeida Roger J., Gerotto E., Zoppellaro G., Tucek J., Safarova K.C., Zboril R. (2017). Electrostatically stabilized hybrids of carbon and maghemite nanoparticles: Electrochemical study and application. Phys. Chem. Chem. Phys..

[52] Magro M., Baratella D., Miotto G., Frömmel J., Šebela M., Kopečná M., Agostinelli E., Vianello F. (2019). Enzyme self-assembly on naked iron oxide nanoparticles for aminoaldehyde biosensing. Amino Acids.

[53] Baratella D., Magro M., Sinigaglia G., Zboril R., Salviulo G., Vianello F. (2013). A glucose biosensor based on surface active maghemite nanoparticles. Biosens. Bioelectron..

[54] Magro M., Baratella D., Colò V., Vallese F., Nicoletto C., Santagata S., Sambo P., Molinari S., Salviulo G., Venerando A. (2019). Electrocatalytic Nanostructured Ferric Tannates as platform for enzyme conjugation: Electrochemical determination of phenolic compounds. Bioelectrochemistry.

[55] Shinde S.B., Fernandes C.B., Patravale V.B. (2012). Recent trends in in-vitro nanodiagnostics for detection of pathogens. J. Control. Release.

[56] Sanvicens N., Pastells C., Pascual N., Marco M.P. (2009). Nanoparticle based biosensors for detection of pathogenic bacteria. TrAC Trends Anal. Chem..

[57] Bonaiuto E., Magro M., Fasolato L., Novelli E., Shams S., Piccirillo A., Bakhshi B., Moghadam T.T., Baratella D., Vianello F. (2018). Versatile nano-platform for tailored immuno-magnetic carriers. Anal. Bioanal. Chem..

[58] Martin N.H., Murphy S.C., Ralyea R.D., Wiedmann M., Boor K.J. (2011). When cheese gets the blues: Pseudomonas fluorescens as the causative agent of cheese spoilage. J. Dairy Sci..

[59] Andreani N.A., Martino M.E., Fasolato L., Carraro L., Montemurro F., Mioni R., Bordin P., Cardazzo B. (2014). Tracking the blue: A MLST approach to characterize the Pseudomonas fluorescens group. Food Microbiol..

[60] Chierici M., Picozzi C., La Spina M.G., Orsi C., Vigentini I., Zambrini V., Foschino R. (2016). Strain diversity of Pseudomonas fluorescens group with potential blue pigment phenotype isolated from dairy products. J. Food Prot..

[61] Andreani N.A., Carraro L., Martino M.E., Fondi M., Fasolato L., Miotto G., Magro M., Vianello F., Cardazzo B. (2015). A genomic and transcriptomic approach to investigate the blue pigment phenotype in *Pseudomonas fluorescens*. Int. J. Food Microbiol..

[62] Andreani N.A., Carraro L., Zhang L., Vos M., Cardazzo B. (2019). Transposon mutagenesis in Pseudomonas fluorescens reveals genes involved in blue pigment production and antioxidant protection. Food Microbiol..

[63] Magro M., Baratella D., Jakubec P., Corraducci V., Fasolato L., Cardazzo B., Novelli E., Zoppellaro G., Zboril R., Vianello F. (2019). H_2_O_2_ Tolerance in Pseudomonas fluorescens: Synergy between Pyoverdine-Iron(III) Complex and a Blue Extracellular Product by a Nanotechnology based Electrochemical Approach. ChemElectroChem..

[64] Kiran-Kumar P., Badarinath V., Halami P. (2008). Isolation of anti-listerial bacteriocin producing Lactococcus lactis CFR-B3 from Beans (Phaseolus vulgaris). Internet J. Microbiol..

[65] Magro M., Bonaiuto E., Baratella D., de Almeida Roger J., Jakubec P., Corraducci V., Tucek J., Malina O., Zboril R., Vianello F. (2016). Electrocatalytic nanostructured ferric tannates: Characterization and application of a polyphenol nanosensor. ChemPhysChem.

[66] De Almeida Roger J., Magro M., Spagnolo S., Bonaiuto E., Baratella D., Fasolato L., Vianello F. (2018). Antimicrobial and magnetically removable tannic acid nanocarrier: A processing aid for Listeria monocytogenes treatment for food industry applications. Food Chem..

[67] Hadjidemetriou M., Al-Ahmady Z., Buggio M., Swift J., Kostarelos K. (2019). A novel scavenging tool for cancer biomarker discovery based on the blood-circulating nanoparticle protein corona. Biomaterials.

[68] Miotto G., Magro M., Terzo M., Zaccarin M., Da Dalt L., Bonaiuto E., Baratella D., Gabai G., Vianello F. (2016). Protein corona as a proteome fingerprint: The example of hidden biomarkers for cow mastitis. Colloids Surf. B Biointerfaces.

[69] Magro M., Zaccarin M., Miotto G., Da Dalt L., Baratella D., Fariselli P., Gabai G., Vianello F. (2018). Analysis of hard protein corona composition on selective iron oxide nanoparticles by MALDI-TOF mass spectrometry: Identification and amplification of a hidden mastitis biomarker in milk proteome. Anal. Bioanal. Chem..

[70] Ferreira M.I., Magro M., Ming L.C., da Silva M.B., Rodrigues L.F.O.S., do Prado D.Z., Bonaiuto E., Baratella D., Lima G.P.P., de Almeida Roger J. (2017). Sustainable production of high purity curcuminoids from Curcuma longa by magnetic nanoparticles: A case study in Brazil. J. Clean. Prod..

[71] Magro M., Esteves Moritz D., Bonaiuto E., Baratella D., Terzo M., Jakubec P., Malina O., Cépe K., de Falcao Aragao G.M., Zboril R. (2016). Citrinin mycotoxin recognition and removal by naked magnetic nanoparticles. Food Chem..

[72] Huang S., Li W., Han P., Zhou X., Cheng J., Wen H., Xue W. (2019). Carbon quantum dots: Synthesis, properties, and sensing applications as a potential clinical analytical method. Anal. Methods.

[73] Dykman L.A., Khlebtsov N.G. (2011). Gold Nanoparticles in Biology and Medicine: Recent Advances and Prospects. Acta Naturae.

[74] Baetke S.C., Lammers T., Kiessling F. (2015). Applications of nanoparticles for diagnosis and therapy of cancer. Br. J. Radiol..

[75] Elsabahy M., Wooley K.L. (2012). Design of polymeric nanoparticles for biomedical delivery applications. Chem. Soc. Rev..

[76] Capeletti L.B., Loiola L.M.D., Picco A.S., da Silva Liberato M., Cardoso M.B. (2018). Silica Nanoparticle Applications in the Biomedical Field. Smart Nanoparticles for Biomedicine.

[77] Shirshahi V., Soltani M. (2015). Solid silica nanoparticles: Applications in molecular imaging. Contrast Media Mol. Imaging.

[78] Wang Y., Zhao Q., Han N., Bai L., Li J., Liu J., Che E., Hu L., Zhang Q., Jiang T. (2015). Mesoporous silica nanoparticles in drug delivery and biomedical applications. Nanomed. Nanotechnol. Biol. Med..

[79] Braun K., Stürzel C.M., Biskupek J., Kaiser U., Kirchhoff F., Lindén M. (2018). Comparison of different cytotoxicity assays for in vitro evaluation of mesoporous silica nanoparticles. Toxicol. In Vitro.

[80] Tamba B.I., Dondas A., Leon M., Neagu A.N., Dodi G., Stefanescu C., Tijani A. (2015). Silica nanoparticles: Preparation, characterization and in vitro/in vivo biodistribution studies. Eur. J. Pharm. Sci..

[81] Colombo M., Carregal-Romero S., Casula M.F., Gutierrez L., Morales M.P., Böhm I.B., Heverhagen J.T., Prosperi D., Parak W.J. (2012). Biological applications of magnetic nanoparticles. Chem. Soc. Rev..

[82] Basso C.R., Crulhas B.P., Magro M., Vianello F., Pedrosa V.A. (2019). A new immunoassay of hybrid nanomater conjugated to aptamers for the detection of dengue virus. Talanta.

[83] Venerando R., Miotto G., Magro M., Dallan M., Baratella D., Bonaiuto E., Zboril R., Vianello F. (2013). Magnetic Nanoparticles with Covalently Bound Self-Assembled Protein Corona for Advanced Biomedical Applications. J. Phys. Chem. C.

[84] Skopalik J., Polakova K., Havrdova M., Justan I., Magro M., Milde D., Knopfova L., Smarda J., Polakova H., Gabrielova E. (2014). Mesenchymal stromal cell labeling by new uncoated superparamagnetic maghemite nanoparticles in comparison with commercial Resovist—An initial in vitro study. Int. J. Nanomed..

[85] Cmiel V., Skopalik J., Polakova K., Solar J., Havrdova M., Milde D., Justan I., Magro M., Starcuk Z., Provaznik I. (2017). Rhodamine bound maghemite as a long-term dual imaging nanoprobe of adipose tissue-derived mesenchymal stromal cells. Eur. Biophys..

[86] Magro M., Campos R., Baratella D., Lima G.P.P., Hola K., Divoky C., Stollberger R., Malina O., Aparicio C., Zoppellaro G. (2014). A Magnetically Drivable Nanovehicle for Curcumin with Antioxidant Capacity and MRI Relaxation Properties. Chem. Eur. J..

[87] Magro M., Martinello T., Bonaiuto E., Gomiero C., Baratella D., Zoppellaro G., Cozza G., Patruno M., Zboril R., Vianello F. (2017). Covalently bound DNA on naked iron oxide nanoparticles: Intelligent colloidal nano-vector for cell transfection. Biochim. Biophys. Acta Gen. Sub..

[88] Venerando A., Magro M., Baratella D., Ugolotti J., Zanin S., Malina O., Zboril R., Lin H., Vianello F. (2019). Biotechnological applications of nanostructured hybrids of polyamine carbon quantum dots and iron oxide nanoparticles. Amino Acids.

[89] Anderson C.J., Bulte J.W., Chen K., Chen X., Khaw B.A., Shokeen M., Wooley K.L., VanBrocklin H.F. (2010). Design of targeted cardiovascular molecular imaging probes. J. Nucl. Med..

[90] Kanwar J.R., Roy K., Kanwar R.K. (2011). Chimeric aptamers in cancer cell-targeted drug delivery. Crit. Rev. Biochem. Mol. Biol..

[91] Kubinova S., Sykova E. (2010). Nanotechnologies in regenerative medicine. Minim. Invasive Ther. Allied Technol..

[92] Kiessling F., Huppert J., Zhang C., Jayapaul J., Zwick S., Woenne E.C., Mueller M.M., Zentgraf H., Eisenhut M., Addadi Y. (2009). RGD-labeled USPIO inhibits adhesion and endocytotic activity of alpha v beta3-integrin-expressing glioma cells and only accumulates in the vascular tumor compartment. Radiology.

[93] Dias A.M., Hussain A., Marcos A.S., Roque A.C. (2011). A biotechnological perspective on the application of iron oxide magnetic colloids modified with polysaccharides. Biotechnol. Adv..

[94] Chemello G., Piccinetti C., Randazzo B., Carnevali O., Maradonna F., Magro M., Bonaiuto E., Vianello F., Pasquaroli S., Radaelli G. (2016). Oxytetracycline delivery in adult female zebrafish by iron oxide nanoparticles. Zebrafish.

[95] Magro M., Baratella D., Bonaiuto E., de Almeida Roger J., Chemello G., Pasquaroli S., Mancini I., Olivotto I., Zoppellaro G., Ugolotti J. (2019). Stealth iron oxide nanoparticles for organotropic drug targeting. Biomacromolecules.

[96] Chemello G., Randazzo B., Zarantoniello M., Fifi A.P., Aversa S., Ballarin C., Radaelli G., Magro M., Olivotto I. (2019). Safety assessment of antibiotic administration by magnetic nanoparticles in in vitro zebrafish liver and intestine cultures. Comp. Biochem. Physiol. C Toxicol. Pharmacol..

[97] Skjolding L.M., Sørensen S.N., Hartmann N.B., Hjorth R., Hansen S.F., Baun A. (2016). A critical review of aquatic ecotoxicity testing of nanoparticles—The quest for disclosing nanoparticle effects. Angew. Chem. Int. Ed..

[98] Magro M., De Liguoro M., Franzago E., Baratella D., Vianello F. (2018). The surface reactivity of iron oxide nanoparticles as a potential hazard for aquatic environments: A study on Daphnia magna adults and embryos. Sci. Rep..

[99] Magro M., Bramuzzo S., Baratella D., Ugolotti J., Zoppellaro G., Chemello G., Olivotto I., Ballarin C., Radaelli G., Arcaro B. (2019). Self-assembly of chlorin-e6 on γ-Fe2O3 nanoparticles: Application for larvicidal activity against Aedes aegypti. J. Photochem. Photobiol. B Biol..

[100] Tang S.C.N., Lo I.M.C. (2013). Magnetic nanoparticles: Essential factors for sustainable environmental applications. Water Res..

[101] Plachtová P., Medříková Z., Zbořil R., Tuček J., Varma R.S., Maršálek B. (2018). Iron and iron oxide nanoparticles synthesized with green tea extract: Differences in ecotoxicological profile and ability to degrade malachite green. ACS Sustain. Chem. Eng..

[102] Markova Z., Novak P., Kaslik J., Plachtova P., Brazdova M., Jancula D., Siskova K.M., Machala L., Marsalek B., Zboril R. (2014). Iron(II,III)-polyphenol complex nanoparticles derived from green tea with remarkable ecotoxicological impact. ACS Sustain. Chem. Eng..

[103] Mwilu S.K., Siska E., Baig R.B.N., Varma R.S., Heithmar E., Rogers K.R. (2014). Separation and measurement of silver nanoparticles and silver ions using magnetic particles. Sci. Total Environ..

[104] Coyne R., Smith P., Moriarty C. (2001). The fate of oxytetracycline in the marine environment of a salmon cage farm, Marine Environment and Health Series No. 3, Marine Institute 2001. Mar. Environ. Health Ser..

[105] Wise R. (2002). Antimicrobial resistance: Priorities for action. J. Antimicrob. Chemother..

[106] De La Torre A., Iglesias I., Carballo M., Ramírez P., Muñoz M.J. (2012). An approach for mapping the vulnerability of European Union soils to antibiotic contamination. Sci. Total Environ..

[107] Rakshit S., Sarkar D., Elzinga E., Punamiya P., Datta R. (2014). Surface complexation of oxytetracycline by magnetite: Effect of solution properties. Vadose Zone J..

[108] Figueroa R.A., Mackay A.A. (2005). Sorption of oxytetracycline to iron oxides and iron oxide-rich soils. Environ. Sci. Technol..

[109] Ihara I., Toyoda K., Beneragama N., Umetsu K. (2009). Magnetic separation of antibiotics by electrochemical magnetic seeding. J. Phys. Conf. Ser..

[110] Xiao L., Li J., Brougham D.F., Fox E.K., Feliu N., Bushmelev A., Schmidt A., Mertens N., Kiessling F., Valldor M. (2011). Water-soluble superparamagnetic magnetite nanoparticles with biocompatible coating for enhanced magnetic resonance imaging. ACS Nano.

[111] Magro M., Baratella D., Molinari S., Venerando A., Salviulo G., Chemello G., Olivotto I., Zoppellaro G., Ugolotti J., Aparicio C. (2019). Biologically safe colloidal suspensions of naked iron oxide nanoparticles for in situ antibiotic suppression. Colloids Surf. B Biointerfaces.

[112] Magro M., Domeneghetti S., Baratella D., Jakubec P., Salviulo G., Bonaiuto E., Venier P., Malina O., Tuček J., Ranc V. (2016). Colloidal Surface Active Maghemite Nanoparticles for biologically safe CrVI remediation: From core-shell nanostructures to pilot plant development. Chem. Eur. J..

[113] Colino C., Millán C., Lanao J. (2018). Nanoparticles for signaling in biodiagnosis and treatment of infectious diseases. Int. J. Mol. Sci..

[114] Cho I.H., Ku S. (2017). Current technical approaches for the early detection of foodborne pathogens: Challenges and opportunities. Int. J. Mol. Sci..

[115] Magro M., Baratella D., Bonaiuto E., de Almeida Roger J., Vianello F. (2017). New Perspectives on Biomedical Applications of Iron Oxide Nanoparticles. Curr. Med. Chem..

[116] Mahmoudi M., Lynch I., Ejtehadi M.R., Monopoli M.P., Baldelli Bombelli F., Laurent S. (2011). Protein−Nanoparticle Interactions: Opportunities and Challenges. Chem. Rev..

[117] Jersmann H.P., Dransfield I., Hart S.P. (2003). Fetuin/Alpha2-HS Glycoprotein Enhances Phagocytosis of Apoptotic Cells and Macropinocytosis by Human Macrophages. Clin. Sci..

[118] Cedervall T., Lynch I., Lindman S., Berggård T., Thulin E., Nilsson H., Dawson K.A., Linse S. (2007). Understanding the nanoparticle–protein corona using methods to quantify exchange rates and affinities of proteins for nanoparticles. Proc. Natl. Acad. Sci. USA.

[119] Garcia J., Zhang Y., Taylor H., Cespedes O., Webb M.E., Zhou D. (2001). Multilayer Enzyme-Conjugated Magnetic Nanoparticles as Efficient, Reusable Biocatalysts and Biosensors. Nanoscale.

[120] Niemirowicz K., Markiewicz K.H., Wilczewska A.Z., Car H. (2012). Magnetic nanoparticles as new diagnostic tools in medicine. Adv. Med. Sci..

[121] Mirshafiee V., Mahmoudi M., Lou K., Cheng J., Kraft M.L. (2013). Protein corona significantly reduces active targeting yield. Chem. Commun..

[122] Salvati A., Pitek A.S., Monopoli M.P., Prapainop K., Bandelli Bombelli F., Hristov D.R., Kelly P.M., Åberg C., Mahon E., Dawson K.A. (2013). Transferrin-functionalized nanoparticles lose their targeting capabilities when a biomolecule corona adsorbs on the surface. Nat. Nanotechnol..

[123] Laurent S., Burtea C., Thirifays C., Rezaee F., Mahmoudi M. (2013). Significance of cell “observer” and protein source in nanobiosciences. J. Colloid Interface Sci..

